# Health resource use and epidemiologic profile of herpes zoster outpatients aged 50 years or older: a modified Delphi consensus panel in Brazil

**DOI:** 10.1016/j.bjid.2025.104560

**Published:** 2025-07-04

**Authors:** Ângela Maria Bagattini, Michelle Quarti Machado da Rosa, Jorge A. Gomez, Jamile Ballivian, Agustín Casarini, Ariel Bardach, Cristiana Maria Toscano

**Affiliations:** a Universidade Federal de Goiás, Instituto de Patologia Tropical e Saúde Pública, Goiânia, GO, Brazil; b Value Evidence & Outcomes, GSK Vaccines, Emerging Markets, Av. del Libertador 7208, CABA C1429BMS, Argentina; c Institute for Clinical Effectiveness and Health Policy, Argentina; d Center for Research in Epidemiology and Public Health, CONICET, Argentina

**Keywords:** Herpes zoster, Post-herpetic neuralgia, Healthcare resource, Epidemiology

## Abstract

Herpes Zoster (HZ) and its complications, such as Postherpetic Neuralgia (PHN), are associated with significant burden in elderly. In Brazil, data on economic and epidemiologic HZ burden is still limited. We conducted a Delphi panel to assess healthcare resource use in HZ outpatients aged 50-years and older. Diagnosis and treatment resources, proportion of referral and hospitalization were estimated considering HZ, PHN, ophthalmic and neurologic zoster typical cases. A diverse group of 20 medical specialists was selected, and responded anonymously to an online questionnaire. Consensus was met if ≥ 75 % agreement was reached in the 1st round, and if not met, a 2nd round was held. Summary statistics are reported stratified by age-groups and healthcare system (public and private). Responses were obtained from 19 and 17 panel members in the 1st and 2nd rounds, respectively. The proportion HZ outpatients with PHN increased significantly with age (4 % in 50‒59; 14 % in ≥ 80 years). Ophtalmic and neurological complications ranged from 5 %‒13 % across age groups. Absenteeism was high, ranging from 30 %‒68 % of patients depending on the clinical presentation. HZ patients required 2‒3 medical visits, and referral to another medical specialty varied from 10 %‒22 % across age ranges, doubling for NPH patients. Proportion of hospitalization varied from 1–8 %. HZ diagnosis was mainly clinical (93 %). Acyclovir (95 %) and valaciclovir (80 %) were the therapy of choice in the public and private systems, respectively. Pain management included dipyrone and codeine (63 %), pregabalin (58 %), and gabapentin (Neurontin) (48 %). Our results report significant healthcare resource utilization by elderly HZ patients in Brazil.

## Introduction

Herpes Zoster (HZ), commonly known as shingles, is a distressing and painful neurocutaneous illness. It is caused by the reactivation of the Varicella-Zoster Virus (VZV) associated with situations of low immunity, including immune senescence and immunosuppression as a result of disease or treatment.^1^^,^^2^ Estimated seroprevalence VZV rates are high rates across countries and increase with age, reaching 90 % in adults.^3^ With the advent of childhood vaccination against VZV, varicella has become a preventable disease and its incidence has significantly decreased. Nonetheless, the incidence of HZ has continued to increase globally.^4^ Almost 30 % of individuals are at risk of developing HZ in their lifetime, with risk increase with age, reaching around 50 % in those aged 80 years and over.^5^^,^^6^

Among populations at high risk in Latin America, the cumulative incidence of HZ ranges from 318–3423 cases per 100,000 persons per year of follow-up. Disease incidence increases significantly after 50 years of age reaching 6–8 and 8–12 per 1000 person-years at 60 and 80 years of age, respectively.^5^ This is consistent with the findings reported in North America, Europe, and Asia-Pacific regions.^7^

Generally, HZ presents itself as a painful, self-limiting unilateral dermatomal rash affecting mainly the thoracic (53 % of cases), followed by the cervical (20 %), trigeminal including ophthalmic (15 %), and lumbosacral regions (11 %). Usually, HZ resolves within a few weeks, but if left untreated, it can lead to serious complications, including secondary bacterial infections, neurological adverse events such as Post-Herpetic Neuralgia (PHN), and ophthalmological adverse events such as keratitis and loss of vision.^8^ PHN, a chronic neuropathic pain, is one of the most common age-associated sequelae, occurring in up to 30 % of cases, while other complications, for example, disseminated cutaneous HZ and Ramsay Hunt syndrome, occur less frequently. The persistent pain associated with PHN has been reported to extend for more than a year in over 30 % of patients, impairing social life and interfering with normal activities.^5^^,^^7^ While deaths due to HZ are infrequent (0.19–0.51 cases per million),^9^ severe cases of HZ and its complications may require hospitalization (3 %–35.7 %).^5^ The disease and its complications are thus associated with reduced quality of life of patients and significant economic burden.^4^

Prompt oral antiviral therapy is recommended for HZ patients, being usually administered for 7days in the absence of complications. Intravenous antiviral is recommended for immunosuppressed patients or those requiring hospitalization and with neurologic or ophthalmic complications. Pain management of acute and PHN events is complex and may require several drugs, including opioids.^10^, ^11^, ^12^

Two HZ vaccines – a live-attenuated VZV vaccine and a recombinant adjuvanted VZV glycoprotein E subunit vaccine – are available for prevention in healthy older adults,^13^ and their use can help reduce HZ related morbidity, particularly in setting with high disease burden.

Brazil’s demographic transition and increasing older population is happening at a rapid pace and by 2030 people aged 60 years and older will represent 24 % (∼50 million) of the total population.^14^ As older adults and elderly are the main risk groups for HZ, this may imply in further increase in disease and economic burden due to HZ and its complications over time. As such, estimates of disease burden, resource use, and disease diagnosis and management are relevant to support a better understanding of disease epidemiology, burden and economic impact in the country.

In Brazil, there is limited information regarding the resource use of HZ patients, particularly outpatients, including diagnostics, therapy including antivirals and pain management, medical visits and referral rates, and proportion of patients with complications and requiring hospitalization. Further, patient management and care vary in the private (National Supplementary Agency) and public (SUS) healthcare systems based on availability and reimbursement of diagnostic and therapeutic technologies recommended by international clinical guidelines. As such, we sought to assess the healthcare resource use of HZ outpatients aged 50 years and older in Brazil via a modified Delphi panel,^15^ considering the public and the private healthcare system, and addressing HZ and its complications.

## Material and methods

### Study design

A modified Delphi panel ‒ a qualitative research tool often used in healthcare to obtain consensus on a specific topic among a panel of experts,^16^ was conducted considering recommendations of Trevelyan 2015^17^ and Nasa 2021,^18^ in order to assess assumptions and obtain consensus in areas where information is limited or lacking.^19^

The study protocol was developed a priori and the Research Ethics Committee of the Federal University of Goiás in Goiania, Brazil, granted ethical approval for this investigation in December 2022 (#5.822.872). Electronically signed consent was obtained from those who agreed to participate, and the participants were reimbursed for their time in the amount of R$500.00.

### Selection of Delphi panel members

Members identified by referral from medical societies, specialists in the field, and research groups were invited to be on the Delphi panel. Accordingly, a diverse group of specialists with experience in HZ care from the public and/or the private healthcare system, in the various levels of care, and from all 5 macro-regions of the country, were invited to participate in the expert panel. There are no standard recommendation with respect to the number of panel/experts and it varies from 10 to 1000. However, most of the studies are conducted with 20 to 50 panelists, and the number may be even smaller when the group is highly homogeneous.^15^^,^^18^ The panel was composed of 20 members representing infectious disease specialists (*n* = 8), dermatologists (*n* = 5), neurologists (*n* = 2), ophthalmologists (*n* = 2), geriatricians/clinicians (*n* = 2), and physiatrists (*n* = 1). Panel members were informed about the objective and procedure of the study and those who agreed to participate were asked to sign an informed consent.

### Data collection

**Fig. 1:**
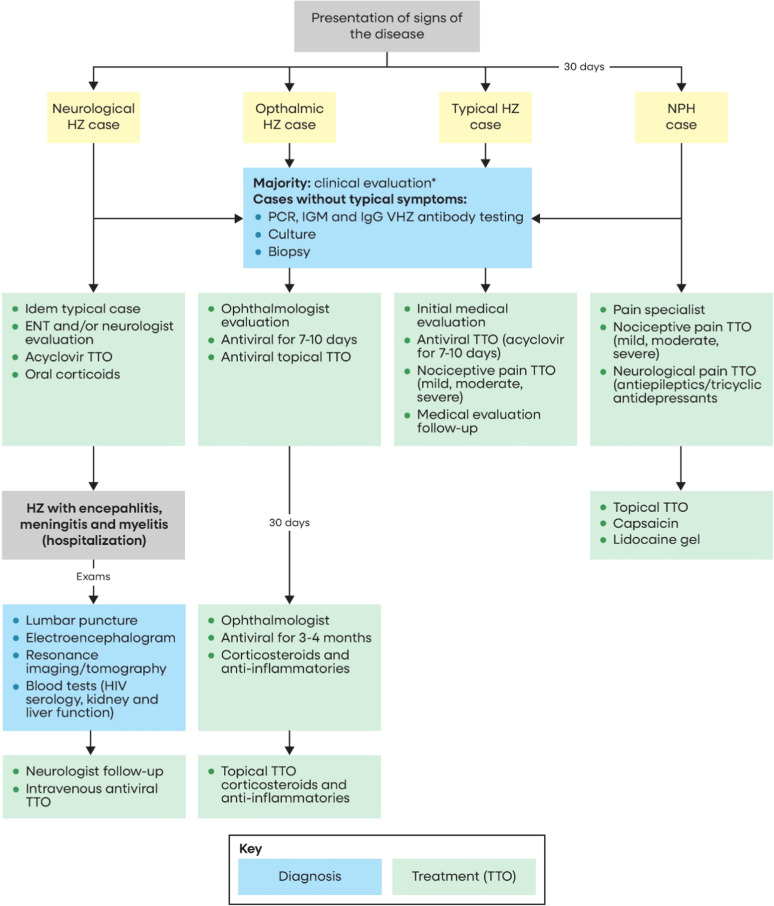
Flowchart Depicting Clinical Presentations of HZ considered in the Delphi Panel, by guideline recommended diagnostic and therapeutic approaches. Brazil, 2023.

The Delphi questionnaire was structured by areas of expertise and considering a variety of relevant clinical presentations including a typical HZ case, PHN case, ophthalmic zoster, and neurologic zoster in immunocompetent individuals (Fig. 1). This flowchart was presented early on the questionnaire to all panel members, and depicted clinical presentations of HZ to be considered when responding to the questionnaire, along with the diagnostic therapeutic approaches recommended by current international guidelines.^10^, ^11^, ^12^^,^^20^, ^21^, ^22^, ^23^, ^24^, ^25^

Parameters and questions included in the questionnaire were defined considering both a literature review conducted in April 2023, and available data from the National health information systems (public and private) in Brazil by the project team. The literature search updated a previous review conducted by Bardach et al. 2021,^5^ replicating terms used and search strategy. Data elements which was not available, limited or inconsistent were considered and included in the Delphi panel assessment.

These included estimates of proportion of HZ patients with PHN or recurrent PHN; duration of HZ and PHN episode; proportion of patients with selected complications (dermatologic, ophthalmic, neurologic, or other complication); proportion of patients requiring referral to other specialties; proportion of patients requiring hospitalization; and proportion of patients reporting work absenteeism, by HZ clinical presentations considered. Absenteeism was defined as the proportion of patients who were away from occupational activities due to prescribed medical leave. Average number of lost days of work was estimated considering the duration of prescribed medical leave.

In addition, diagnostic and therapeutic management of patients was also assessed, diagnostic modalities (clinical only, clinical and laboratory) and types of laboratory diagnostic tests used; average time of medical follow up; laboratory exams and medical visits during patient follow up; type of antivirals and number of days used; therapy for pain management and number of days used, and other medications, also by HZ clinical presentations considered.

Questions were stratified by public vs. private healthcare system and age group (50‒59, 60‒69, 70‒79 and 80+ years of age).

Questionnaire was pilot tested with the project team and a guest medical expert. The online questionnaire was structured and made available via SurveyMonkey®. Questionnaires were comprised of 140 and 69 questions, in the 1st and 2nd Delphi panel rounds, respectively.

All participants who agreed and signed the informed consent were sent the online questionnaire and were given 20 days to fill out and reply to their responses. A reminder was sent to panel members 4 days before the deadline. Responses was anonymous. All panel members were asked to respond to the questionnaire according to their expertise. Infectious diseases specialists, dermatologists, and geriatricians/clinicians provided answers regarding the typical case of HZ and PHN. They also provided answers for the neurological and ophthalmic cases if they had experience with these. Similarly, neurologists provided answers related to PHN and the neurological case, while ophthalmologists responded only to the ophthalmic case.

To answer the questions, experts were asked to consider a population of 100 patients with HZ and indicate the average proportion of patients affected by the simulated scenarios or the average number of resources used in each described situation. Panel members were also provided with the opportunity to comment and propose new items if needed.

### Consensus process

As it was required that a consensus be reached, two rounds of the Delphi panel took place. After responding to the questionnaire, panel members received a summary of the group replies based on which individual experts modified their responses. This process was repeated until an expert consensus was reached. Consensus was defined as a percentage of agreement based on a predefined cut-off value usually selected arbitrarily^18^ – which was defined as 75 % or more for the 1st round. If there was no consensus in the 1st round, another round was held and a new questionnaire considering only questions for parameters to which no consensus was reached was sent to panel members.

Consensus threshold was defined as 70 % or more in the 2nd round. Questions were presented with reported average values from the literature (when available) or the average and minimum/maximum values obtained from the 1st round of the panel. Response options were presented with a 4-point Likert-type scale (1 = strongly disagree, 2 = partial disagree, 3 = partial agree, and 4 = strongly agree). The experts had the chance to reflect on their previous responses in light of these scores and to revise them when they disagreed with the midpoint. If consensus was not reached in the 2nd round, another round could be held to explore the group’s divergence points.

### Data analysis

Descriptive analysis was conducted, and summary statistical measures are reported as average and minimum and maximum values for numerical variables, and percentages for proportions. Results were stratified by age-groups and healthcare system (public and private). For consensus assessment, inter-respondent agreement was calculated considering intra class correlation coefficient. If 1–2 panelists differed in their answers in relation to the other panelists by > 50 % with extreme values, their experience was assessed considering number of HZ patients they had treated per year and their answers were excluded if they had no significant experience with that specific issue.

## Results

### Panel characteristics and response rates

Among the 20 experts who agreed to participate in the 1st round of the Delphi panel, 19 completed the questionnaire. For the 2nd round, only the respondents of the 1st round were considered, and 17 experts completed the questionnaire. The detailed steps, procedures and timeline of the Delphi panel are presented in Supplement Table 1, whereas the characteristics of Delphi Panel members, by geographic location, level of care and type of healthcare system where they provide care are presented in Table 1. Over 50 % of the panel members were from the Southeast region of Brazil. Panel members reported a range of 3 to 50 HZ patients treated in the previous year. The average time for questionnaire completion was 60 and 28 minutes in the 1st and 2nd rounds, respectively.

**Table 1:** Characteristics of Delphi panel members, by geographic macro-region, level of care and type of healthcare system. Brazil, 2023.

Characteristics	n (%)
Geographic Macro-Region of Work	
North	1 (5)
Northeast	1 (5)
Center-West	5 (26)
Southeast	10 (53)
South	2 (11)
Level of Care^a^ where Services are Provided	
Primary care	14 (74)
Secondary (outpatient and hospital) care	7 (37)
Tertiary care	9 (47)
Health sector where Services are Provided	
Public	3 (16)
Private	4 (21)
Both	12 (63)

n, number.

a Some specialists work in more than one sector.

### Estimates of HZ disease progression, complications, and work absenteeism

Fig. 1 depicts clinical presentations of HZ considered in the Delphi Panel. Parameters of disease progression, complications and work absenteeism are presented in Table 2. The proportion of patients with episodes of PHN and PHN recurrence increased with age, from 4 % and 3 %, respectively, for the 50–59 age group, to 14 % for the 80 and over age group. Similar observations were noted for patients presenting with dermatological and ophthalmic complications.

**Table 2:** HZ disease progression, complications, and work absenteeism estimated by the Delphi panel, by age group, for both public and private health care systems. Brazil, 2023.

	50–59 years old	60–69 years old	70–79 years old	80+ years old
	Average (Min‒Max)	Average (Min‒Max)	Average (Min‒Max)	Average (Min‒Max)
**HZ patients progressing to PHN (%)**	4 (3–8)	6 (5–12)	11 (10–20)	14 (13–28)
**HZ patients with reported PHN recurrence (%)**	3 (3–4)	5 (5–6)	13 (10–20)	14 (8–20)
**HZ patients with dermatological complications (%)**	5 (4–20)	10 (9–18)	12 (11–18)	13 (12–16)
**HZ patients with neurological complications (%)**	1 (1–1)	1 (1–1)	1 (1–1)	4 (2–5)
**HZ patients with ophthalmic complications (%)**	4 (4–7)	9 (9–9)	11 (11–12)	12 (12–16)
**HZ patients with other complications (%)**	1 (1–5)	1 (1–1)	3 (2–3)	2 (2–2)
**HZ patients reporting work absenteeism (%)**	50			
**Number of days absent from work (for those reporting absenteeism due to HZ) (n)**	29.1 (14–56)			
**PHN patients reporting work absenteeism (%)**	30			
**Number of days absent from work (for those reporting absenteeism due to PHN) (n)**	39 (39–39)			
**Patients with ophthalmic HZ reporting work absenteeism (%)**	68			
**Number of days absent from work (for those reporting absenteeism due to ophthalmic HZ) (n)**	18 (14–21)			
**Patients with neurological HZ reporting work absenteeism (%)**	48			
**Number of days absent from work (for those reporting absenteism due to neurological HZ) (n)**	39 (39–39)			

HZ, Herpes Zoster; n, number; PHN, Post-Herpetic Neuralgia, y, years; Min, Minimum; Max, maximum.

Across age groups, the proportion of patients absent from work due to HZ and PHN was 50 % and 30 %, respectively. Among these, the average number of workdays lost were 29 and 39 due to HZ and PHN, respectively. These estimates increased in patients with complicated zoster, reaching 68 % in patients with ophthalmic complications, and 48 % with neurological complications. The number of additional days of work lost due to these complications was 18 and 39, respectively. Absenteeism estimates did not differ by age groups.

### Healthcare resources use by HZ outpatients

Table 3 shows the number of consultations in outpatient care and subsequent proportion hospital admissions for a standard case of HZ and PHN in the private healthcare system as determined by the Delphi panel. The number of medical consultations for HZ and PHN varied from 2 to 3 and 4 to 6 increasing with age group, respectively, and did not differ significantly by healthcare system. The proportion of patients referred to another medical specialty also increased with age and was higher in private when compared to patients in the public healthcare system. The proportion of patients requiring hospitalizations increased with age from 1 % for the 50–59 age group to 8 % for the 80 and over age group in both health systems.

**Table 3:** Healthcare service utilization ‒ medical visits, referral and hospitalization of for HZ and PHN outpatients, by age group and healthcare system. Brazil, 2023.

	Age group (in years)	HZ		PHN	
		Average (min–max)		Average (min–max)	
		Private	SUS	Private	SUS
**Medical appointments (n)**	50–59	2 (1–2)	2 (2)	4 (2–4)	3 (2–4)
	60–69	2 (1–2)	2 (2–3)	5 (3–5)	4 (3–5)
	70–79	3 (1–3)	3 (3)	5 (5–6)	4 (3–5)
	80+	3 (3–5)	3 (2–3)	6 (6–6)	5 (3–6)
**Proportion of patients referred to another medical specialty (%)**	50–59	10 (3–48)	8 (3–28)	32 (0–98)	21 (0–68)
	60–69	15 (3–48)	10 (3–28)	37 (3–98)	26 (3–78)
	70–79	29 (3–78)	19 (3–78)	51 (13–98)	40 (3–98)
	80+	33 (3–78)	21 (3–78)	55 (13–98)	41 (3–98)
**Proportion of patients requiring hospitalization (%)**	50–59	1 (1–3)	1 (1)	1 (1–1)	2 (1–3)
	60–69	2 (2–6)	3 (2–8)	2 (2–2)	3 (2–4)
	70–79	4 (3–9)	4 (3–10)	4 (3–5)	4 (3–5)
	80+	8 (6–8)	8 (4–15)	8 (8–8)	8 (8)

HZ, Herpes Zoster; n, number; PHN, Post-Herpetic Neuralgia.

Regarding HZ diagnosis and clinical management, main types of resources used in the private and public healthcare systems are presented in Table 4. HZ diagnosis was predominantly confirmed by clinical criteria in more than 90 % of HZ typical cases in both public and private healthcare systems. Regarding antiviral use, typical HZ patients are predominantly treated with acyclovir (95 %) public health system, while in the private sector valacyclovir is mostly used (80 %).

**Table 4:** Diagnostics and Therapeutics for HZ and PHN outpatients, by healthcare system. Brazil, 2023.

Age group (in years)	HZ		PHN	
	Proportion of patients (%)		Proportion of patients (%)	
	Private	SUS	Private	SUS
Diagnostic procedures/tests				
Clinical only^a^	93 %	95 %	76 %	76 %
Laboratory + clinical^b^	7 %	5 %	24 %	24 %
For those using laboratory procedures, types of tests used:				
Polymerase chain reaction (PCR)	50 %	20 %	0 %	0 %
Herpes zoster – IgG, IgM dosage	14 %	30 %	0 %	0 %
Tzank smear (cytological for herpes virus)	36 %	50 %	0 %	0 %
Skin biopsy	0 %	0 %	30 %	23 %
Therapy – Antivirals and Pain Management				
*Antivirals*				
Acyclovir	15 %	95 %	0 %	0 %
Famciclovir^a^	5 %	1 %	0 %	0 %
Valaciclovir	80 %	4 %	0 %	0 %
*Pain Management*				
Diclofenac	1 %	2 %	0 %	0 %
Dipyrone	63 %	64 %	0 %	0 %
Ibuprofen	6 %	11 %	0 %	0 %
Prednisone	36 %	29 %	0 %	0 %
Codeine	63 %	60 %	36 %	42 %
Tramadol	32 %	34 %	35 %	36 %
Morphine	2 %	1 %	5 %	6 %
Methadone	NU	NU	5 %	6 %
Amitriptyline	NU	NU	40 %	56 %
Carbazepine	NU	NU	16 %	27 %
gabapentin (Neurontin)	NU	NU	48 %	45 %
Pregabalin	NU	NU	58 %	27 %
Capsaicin	NU	NU	11 %	2 %
Lidocaine	NU	NU	14 %	9 %
Nerve blocks for pain^a^	NU	NU	10 %	4 %
Corticosteroids (topical agent)	NU	NU	NU	NU

NU, Not Used.

a For diagnosis we asked the proportion of patients in whom the diagnosis is: exclusively clinical versus clinical + laboratory exams.

b The clinical diagnosis includes the medical appointment which was already accounted. For those in whom the diagnosis is clinical + laboratory exams, we asked the proportion of the exams.

Pain management was initially addressed with dipyrone and codeine for around 60 % of typical HZ cases, while prednisone and tramadol were used in 30 % of cases. For PHN cases, the use of amitriptyline was reported in 56 % of patients in the public healthcare system and 40 % of patients in the private system, followed by gabapentin (Neurontin) in around 45 % of patients in both systems. The use of codeine and tramadol was also mentioned in PHN for up to 42 % and 36 % of cases, respectively. Pregabalin was indicated in 27 % and 58 % of patients from the public and private healthcare systems respectively (Table 4).

## Discussion

Different forms of consensus among experts have been used in situations of lack or limited availability of evidence,^15^ and among these the Delphi panel technique is the most widely used in the context of health technology assessments.^26^ To our knowledge, this is the first study that uses the modified Delphi consensus for estimating healthcare resource utilization in HZ outpatient care in Brazil.

The expert group indicated that 4 % to 14 % of patients with HZ develop PHN, varying by age and with highest proportion in those aged 80 years and over. This estimated proportion is consistent and within the estimated range of estimates reported by studies conducted in other countries. For example, in a prospective cohort study within Kaiser Permanente Northern California, the percentage of HZ cases with PHN increased with age from 2.9 % in persons aged 50–59 years to 12.5 % in persons aged 80 years or older.^27^ In another study, the prevalence of PHN among chronic pain patients receiving treatment in a pain or general clinic or hospital in Brazil was 3.3 %.^28^ In a pooled analysis of prospective cohort studies of HZ patients aged over 50-years from North America, Latin America, and Asia, one-fifth (21.1 %) of HZ patients developed PHN.^29^

Other unusual but severe complications of HZ include ophthalmic, neurological, and dermatological conditions, the incidence of which in many regions is rarely reported.^25^^,^^30^ According to members of the panel in this study, the proportion of patients with such complications ranged from 1 % to 12 % across age groups, also increasing with extreme ages.

Studies have demonstrated that management of HZ patients with or without complications accounts for significant healthcare resource utilization.^31^^,^^32^ According to the panelists, the number of outpatient consultations per HZ episode ranged from 2 to 3, varying by age group, but consistent in both healthcare systems. Also, the number of outpatient visits for PHN patients increased with age (4–6 visits) and were almost double the values for HZ patients in all age groups. These results are similar to previously reported in Brazil, where a prospective observational study estimated an average of 3.5 outpatient medical visits per HZ case.^33^ Similarly, in the US a study of medical insurance claims, the number of outpatient visits ranged from 2 to 3, varying with age.^24^ Further, in the present study, the frequency of hospitalizations for HZ/PHN ranged from 1 % to 8 % in the age groups from 50–59 to over 80-years-old. This result is comparable to the 5.7 % hospitalization of HZ patients reported from a pooled analysis of studies in Argentina, Brazil, and Mexico.^34^ Further, the number of days absent due to HZ and PHN in the present study was 29 and 39, respectively. HZ is known to have a negative effect on productivity and is responsible for work absences.^35^ In a prospective study, 19.9 % of HZ patients missed almost 45 full workdays due to HZ associated disease burden.^33^

The identification of the medical resources used by the specialists is essential to estimate the costs of patient management including diagnosis and treatment, particularly as there is no specific guideline for the treatment of HZ in Brazil. With a combination of public and private healthcare systems,^36^ with private health insurance providing services to approximately 30 % of the country’s population,^37^ it is important to evaluate any difference in healthcare resource use among these two complementary healthcare systems. Our results also reflect HZ outpatient diagnosis and treatment patterns in both the public and private systems. To this end, our data showed some differences in practice between private and public care, such as the predominant use of acyclovir in the public system and valacyclovir in the private system, and a higher proportion of patients referred to hospitalizations in the private system.

Several limitations to our estimates should be noted. First, the use of consensus from expert’s opinion is graded as the lowest level of evidence and with the highest risk of bias.^38^ Nonetheless, some tools have supported improvements in the use of consensus in healthcare for a more transparent and complete reporting of data, such as the ACCORD (ACcurate COnsensus Reporting Document) guideline.^39^ We have complied to these guidelines, minimizing some of the bias inherent to the Delphi methodology. These include including experts based on their clinical practice, representing all regions of Brazil as panel members, and offering anonymity of responses.

Other limitations include the fact that not all experts participated in all Delphi rounds, the variability of clinical experience in terms of the number of patients treated of panel members, and the varying clinical approaches by medical specialty reported by the panel members. Finally, medical professionals from different regions and working in different settings may reflect variations in healthcare structures and resources available. Despite these limitations, there is value in using methodologies such as the Delphi panel.^40^

Limited evidence is available on the disease and economic burden of HZ globally. Our results tackle some of these gaps, particularly related to HZ outpatient resource use in Brazil, demonstrating a significant proportion and recurrence of PHN increasing with age. Notably, healthcare resource use, referrals, complications and more severe outcomes of HZ, such as PHN are particularly high in those aged 80 and above. This data underscores the importance of defining diagnostic and clinical guidelines for HZ management in Brazil, and also to inform disease and economic burden estimates which will be relevant to support healthcare decision makers regarding HZ prevention and management strategies, and plan healthcare service and resource allocation in both the public and the private healthcare systems. SAGE recommended that the use of the recombinant herpes zoster vaccine in a 2-dose schedule with a minimum 2-month interval between doses, for the prevention of herpes zoster in older adults, those with chronic conditions and the immunocompromised, be considered in countries where herpes zoster is an important public health problem. SAGE advised countries to conduct cost-effectiveness analyses to inform decision-making.^41^

## Conflicts of interest

AMB, MQMR and CMT conducted this study as part of their institutional activities at the Research Support Foundation – Federal University of Goias (FUNAPE-UFG) and JB, AC and AB conducted this study as part of their institutional activities at IECS. GSK funded the institutions, which managed both the project and their compensation. Jorge Gomez is employed and hold financial equities in GSK..Authors disclose no other financial and non-financial interest.

## Appendix Supplementary materials

Image, application 1
