# Supplementary material for: Health resource use and epidemiologic profile of herpes zoster outpatients aged 50 years or older: a modified Delphi consensus panel in Brazil
Source: Braz J Infect Dis. 2025 Jul 4;29(5):104560. doi: 10.1016/j.bjid.2025.104560 (PMC12271774; doi:10.1016/j.bjid.2025.104560)
Supplement: Supplementary file 1 [file mmc1.docx]

**BJID-D-25-00033_ Supplementary Material**

**Supplement Table 1** Delphi Panel Deliverables, Steps and Procedures over time. Brazil, 2023.

| **Deliverables** | **Description** | **Schedule** |
| --- | --- | --- |
| 1^st^ round questionnaire | Questionnaire preparation | May 28, 2023 |
| Review draft 1 | Project team discussed and suggested additions to the questionnaire | May 30, 2023 |
| Questionnaire/electronic form 1^st^ round Delphi draft 2 | Questionnaire/electronic form review and development | June 7, 2023 |
| Review draft 2 (team UFG + IECS) | Project team discussed and suggested additions to the questionnaire | June 7 to June 14, 2023 |
| Questionnaire/electronic form 1^st^ round Delphi draft 3 | Questionnaire/electronic form review and development | June 16, 2023 |
| Review draft 3 | Project team discussed and suggested additions to the questionnaire | June 21, 2023 |
| Questionnaire/electronic form 1^st^ round Delphi pilot | Questionnaire/electronic form review and development | June 23, 2023 |
| Final review (team UFG + IECS) | Project team discussed and suggested additions to the questionnaire | June 27, 2023 |
| **Pilot testing of questionnaire/electronic form** | Questionnaire validation with an ad hoc expert | July 7 to July 18, 2023 |
| **Panel member identification** | Coordination team identified potential panel members | June to July, 2023 |
| Assessing participation interest | Checking the interest of members to participate in the Delphi | July 2023 |
| **Invitation to participate** | Coordination team sent official invitation and electronic individual consent form | July 2023 |
| 1^st^ round Delphi panel | Questionnaire sent to panel members who agreed to participate and signed the consent form | July 31 to August 20, 2023 |
| 1^st^ round Delphi panel | Reminder sent via an email for reply (7 and 4 days before the deadline) | August 12 and 16, 2023 |
| 1^st^ round Delphi panel | Summary data of 1^st^ round | August 20 to August 31, 2023 |
| 2^nd^ round Delphi panel | Submission of a new questionnaire or web conference for questions that had no agreement (< 75%) | September 7 to September 18, 2023 |
| 2^nd^ round Delphi panel | Email sent reminding of the deadline for replying or the limit for holding the web conference | September 11, 2023 |
| 2^nd^ round Delphi panel | Summary data and analysis of the 2^nd^ round (agreement > 70%, 3^rd^ round not necessary) | September 29, 2023 |
| Delphi panel completion | Data analysis of Delphi panel | October 27, 2023 |

IECS, Institute for Clinical Effectiveness and Health Policy; UFG, Universidade Federal de Goiás.
